# Regulating Phase Transition in Neurodegenerative Diseases by Nuclear Import Receptors

**DOI:** 10.3390/biology11071009

**Published:** 2022-07-04

**Authors:** Amandeep Girdhar, Lin Guo

**Affiliations:** Department of Biochemistry and Molecular Biology, Thomas Jefferson University, Philadelphia, PA 19107, USA; amandeep.girdhar@jefferson.edu

**Keywords:** liquid–liquid phase separation, FUS, TDP-43, Kapβ2, ALS, nuclear import receptor

## Abstract

**Simple Summary:**

Aberrant protein phase transitions and aggregation trigger various neurodegenerative diseases, including ALS, FTD, Alzheimer’s disease, and Huntington’s disease. Dynamic exchange of proteins and RNA between the nucleus and cytoplasm is essential for cellular functioning, which is greatly affected by the mislocalization, phase separation, and aggregation of different RNA-binding proteins (RBPs), such as FUS, TDP-43, and hnRNPA1/A2 in their respective neurodegenerative diseases. Disease-linked abnormal accumulation of these RBPs impairs the nucleocytoplasmic transport (NCT) by disrupting the nuclear envelope architecture and mislocalizing essential NCT factors, such as nucleoporins, importins, exportins, and the Ran protein and its regulatory components. Nuclear import receptors (NIRs) have the potential to restore abnormal mislocalization of RBPs by regulating the import/export pathway. NIRs can also potentially prevent or reverse the phase separation and fibrillization of RBPs. Overall, this review focuses on the role of nuclear import receptors in regulating phase transitions in neurodegenerative diseases.

**Abstract:**

RNA-binding proteins (RBPs) with a low-complexity prion-like domain (PLD) can undergo aberrant phase transitions and have been implicated in neurodegenerative diseases such as ALS and FTD. Several nuclear RBPs mislocalize to cytoplasmic inclusions in disease conditions. Impairment in nucleocytoplasmic transport is another major event observed in ageing and in neurodegenerative disorders. Nuclear import receptors (NIRs) regulate the nucleocytoplasmic transport of different RBPs bearing a nuclear localization signal by restoring their nuclear localization. NIRs can also specifically dissolve or prevent the aggregation and liquid–liquid phase separation of wild-type or disease-linked mutant RBPs, due to their chaperoning activity. This review focuses on the LLPS of intrinsically disordered proteins and the role of NIRs in regulating LLPS in neurodegeneration. This review also discusses the implication of NIRs as therapeutic agents in neurogenerative diseases.

## 1. Introduction

Liquid–liquid phase separation (LLPS) is an indispensable and pervasive phenomenon underlying the formation of membraneless organelles and biological condensates throughout the cells [1,2]. A growing number of studies have indicated that these membraneless organelles and condensates are of key importance for normal cellular functions, suggesting a role for LLPS in human health and diseases [3]. Proteins with modular domains and intrinsically disordered regions (IDRs) with low sequence complexity can undergo LLPS and have been shown to promote pathological aggregation in neurodegenerative diseases, such as amyotrophic lateral sclerosis (ALS), frontotemporal lobar degeneration (FTLD), Alzheimer’s disease (AD) and Parkinson’s disease (PD) [4,5,6]. Multiple reports have indicated the pathological role of RNA-binding proteins (RBPs), including TDP-43, FUS, TIA-1, and hnRNPs, in neurodegenerative disorders. RBPs’ cytoplasmic mislocalization, stress granule dynamics dysfunction, and enhanced propensity to aggregate mutant RBPs leads to neurotoxicity [7]. In addition, dysregulation in RBPs function and RNA homeostasis in cells leads to the formation and maturation of biological condensates into more irreversible assemblies and neurotoxic amyloid-aggregates [7]. In several IDRs, the sequence and number of specific amino-acid motifs that facilitate LLPS have been characterized, including tyrosine, glycine, and arginine residues in FUS, hnRNPA1, and Ddx4, multivalent FG repeats in nucleoporins, and (G/S)-(F/Y)-(G/S) LLPS motifs in TDP-43 [3,8,9,10,11]. In these proteins, the formation of multivalent connections facilitated by the presence of dozens of these motifs is instrumental in forming reversible physical cross-links via π–π, cation–π, and sp2–π interactions [8,12]. The LLPS of these proteins is also strongly affected by salt and RNA concentration [13,14]. Disease-causing mutations in RBPs can also accelerate the maturation of droplets to form solid aggregates, which are closely linked to neurodegeneration [15,16,17,18,19]. The transition from phase-separated droplets to solid or gel-phase droplets is controlled by the cells, according to their specific cellular needs [20,21]. The biological factors that control or reverse these phase separation events are not completely known. Several RBPs, including FUS, hnRNPA1, hnRNPA2, TAF15, EWSR1 bearing proline-tyrosine-nuclear localization signal (PY-NLS), and TDP-43 with a canonical NLS (cNLS), mislocalize to cytoplasmic inclusions in neurodegenerative disorders [22]. Several studies have shown that nuclear-import receptors (NIRs) are able to chaperone and disaggregate these disease-linked proteins by engaging their PY-NLSs to restore their nuclear localization and mitigate neurodegeneration [22,23,24,25]. In addition, dysregulation in nucleocytoplasmic transport (NCT) has been reported in ageing and age-related neurodegenerative diseases [26,27]. This review focuses on the role of NIRs in regulating LLPS and NCT and their dysregulation in neurodegeneration. 

## 2. Components and Mechanism of Nucleocytoplasmic Transport

**Figure 1:**
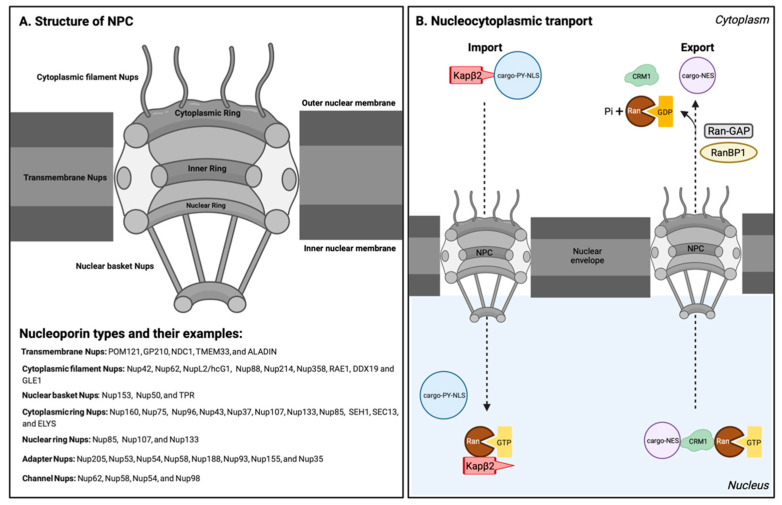
Structure of the NPC and nucleocytoplasmic transport pathway. (**A**) Schematic representation of 3D architecture of the nuclear pore complex (NPC). The major components of the NPC are different types of Nups, as shown in the figure with examples. (**B**) Model for nuclear import using Kapβ2 as an example (**left**), and model for nuclear export using CRM1 as an example (**right**). During the import process, Kapβ2 recognizes PY-NLS-bearing cargo in the cytoplasm. After entering the nucleus, Kapβ2 binds to RanGTP and releases the cargo. During nuclear export, CRM1 binds to cargo in the complex with RanGTP inside the nucleus and transports the complex across the NPC. In the cytoplasm, RanGTP hydrolysis to RanGDP by Ran, with its GTPase activity activated by RanGAP and RanBP1, disassembles the CRM1-cargo complex and releases the cargo. This figure was created with BioRender.com.

Nucleocytoplasmic transport is a bidirectional communication between nucleus and cytoplasm required for the transport of different proteins, RNAs, and ribonucleoproteins [28]. A complicated interplay among varied components of nuclear transport machinery, especially nuclear pore complexes, nuclear envelopes, and numerous soluble transport receptors, regulates NCT. The nuclear pore complex (NPC) is the primary gate for transport between nucleus and cytoplasm [29]. The NPC provides an aqueous channel that permits ions, metabolites, and small proteins with a molecular mass of less than 40 K to 60 K to diffuse. The NPC uses energy-dependent mechanisms to selectively transport particles with diameters of up to 26nm to 28nm [30,31]. The NPC has a cylindrical structure with eight-fold rotational symmetry. It consists of an inner ring positioned between a cytoplasmic ring and a nuclear ring (Figure 1A). Eight fibrils or cytoplasmic filaments decorate the cytoplasmic ring, and a basket-like structure is linked to the nuclear ring [32,33,34,35,36]. In vertebrates, a fully assembled NPC consists of numerous copies of around 30 distinct nucleoporins (Nups) with a molecular mass of ~120 MDa [37]. 

Nups are categorized based on their location within the NPC (Figure 1A) [28]. Transmembrane Nups facilitate NPC formation and nuclear envelope anchoring. Cytoplasmic filament Nups, such as GLE1, interact with the NCT machinery. Nuclear basket Nups are involved in nuclear transport processes. Nups such as Nup98 shuttle in and out of the nucleus and connect with cytoplasmic and nuclear structures [28]. Outer-ring Nups, including cytoplasmic-ring Nups and nuclear-ring Nups, form a complex that is essential for the early stages of nuclear pore complex formation. The inner ring, which harbors the central transport channel and the diffusion barrier of the NPC, is composed of adaptor Nups and channel Nups. Adapter Nups, such as Nup155, which maintains proper protein localization in the inner nuclear membrane, play a critical role in forming the sub-compartments in the nuclear envelope. Nups that contain phenylalanine–glycine (FG) repeats, such as Nup62, which is in the central channel of the inner ring, are called channel Nups and form a hydrogel meshwork within the nuclear pore. FG repeats in Nup proteins are intrinsically disordered domains that are an essential component of the nuclear permeability barrier [29]. FG-Nups regulate the transport of macromolecules, including RNAs and proteins, across the NPC in coordination with nuclear transport receptors (NTRs) [38]. 

Nuclear transport receptors belong to a protein family called karyopherins (Kaps) that includes importins and exportins [39]. Importins, also known as nuclear import receptors, can recognize a nuclear localization signal (NLS) in cargos transported from the cytoplasm to the nucleus; exportins can recognize the nuclear export signal (NES) in cargos transported from the nucleus to the cytoplasm. These Kaps work in combination with their direct interaction with the FG-repeats of Nups in the nuclear pore complexes to mediate nuclear transport [39,40]. 

The Ran protein, which is a member of the Ras superfamily of small GTPases, plays a significant role in nucleocytoplasmic transport. Ran is highly ubiquitous and found in two forms in mammals: Ran guanosine diphosphate (RanGDP) and Ran guanosine triphosphate (RanGTP). GTP hydrolysis by Ran to form RanGDP from RanGTP is responsible for a large portion of the energy required for nucleocytoplasmic transport. Moreover, Kaps associate with RanGTP or RanGDP to regulate the directionality of nucleocytoplasmic transport [39,41,42]. Specifically, the regulator of chromosome condensation 1 (RCC1), which is a guanine nucleotide exchange factor for Ran (Ran-GEF), is found in the nucleus and promotes the transition from RanGDP to RanGTP. On the other hand, the Ran GTPase-activating protein (Ran-GAP) and Ran-binding proteins (RanBPs), which catalyze the hydrolysis of GTP to GDP, are found only in the cytoplasm (Figure 1B) [43,44,45,46]. As a result of this arrangement, RanGTP is concentrated in the nucleus, while RanGDP is concentrated in the cytoplasm, forming a gradient that facilitates nucleocytoplasmic transport with directionality, as discussed in more detail in the following sections on nuclear import and nuclear export, respectively.

### 2.1. Nuclear Import of Proteins with Nuclear Localization Signal by NIR

A nuclear localization signal (NLS) is a short peptide sequence of positively charged amino acids, including lysine and arginine, that acts as a signal peptide to mediate the transport of proteins from cytoplasm to the nucleus [39,47]. NIRs are known to recognize NLS on their cargo proteins to facilitate nuclear import [48]. According to their amino acid composition, NLSs are categorized into classical (cNLS), non-classical (ncNLS), and other types of NLS (Table 1) [47]. The cNLSs are further divided into two categories, “monopartite” (MP) and “bipartite” (BP) (Table 1) [49]. Monopartite NLSs have a single cluster of four to eight basic amino acids which generally contains four or more positively charged amino acids, such as arginine or lysine [49,50]. The bipartite NLSs have two positively charged amino-acid sequences separated by a 9–12 amino-acid linker region (spacer sequence), such as the NLS in TDP-43 [49,50]. Many proteins, including hnRNPA1, FUS, and TAF15, have other types of unusual non-classical nuclear localization signals (ncNLSs), called proline-tyrosine NLSs (PY-NLSs) (Table 1) [47]. PY-NLSs are characterized by 20 to 30 amino acids with N-terminal hydrophobic or basic motifs and C-terminal PY motifs [47,51]. Mutations and post-translational modifications in the NLS sequence of cargo proteins can lead to defects in their nuclear transport [22,24,25].

Signal-umpired nuclear localization is mediated by NIRs that are members of the karyopherin family of proteins, including importin αs (or Kapαs) and importin βs (or Kapβs). Importin αs are adapter proteins that connect cNLS-containing proteins with importin β for nuclear import [84]. Importin α (impα) consists of an N-terminal importin β-binding (IBB) domain, 10 armadillo (Arm) repeats consisting of hydrophobic sequences of approximately 42 to 43 amino acids that function as cNLS-binding sites, and a C-terminal importin α nuclear export factor binding region [85]. Human genome encodes seven importin αs that have been divided into three subfamilies: the α1 subfamily (importin α5, α6 and α7), the α2 subfamily (importin α1 and α8), and the α3 subfamily (importin α3 and α4) [86]. 

Importin βs are another family of nuclear transport receptors that include importins, exportins, and bidirectional receptors [87,88]. There are 20 family members of importin βs in humans that actively participate in the NCT of proteins and RNA [89]. Importin β1 (imp β1) was the first carrier protein identified as importing cNLS-bearing cargoes, such as TDP-43, in combination with importin α [90,91]. On the other hand, Kapβ2 (or importin β2) can recognize a cargo-bearing PY-NLS sequence and regulate nuclear import [92]. Importin βs are flexible, superhelical proteins that typically include 19 to 20 HEAT repeats in a row, each of which is made up of two antiparallel alpha helices, A and B, joined by an acidic loop [40,93]. The N-terminal HEAT repeats bind to Ran, while the C-terminal HEAT repeats bind to IBB or NLS [40,92,94]. 

Ran gradient across the nuclear membrane regulates the directionality of nuclear transport [39,41,42]. For example, Kapβ2, which a low affinity for RanGDP, can bind cargo with PY-NLS inside cytoplasm, where RanGDP is the dominant species. Then, Kapβ2 with PY-NLS-bearing cargo enters the nucleus, where it undergoes a conformation rearrangement after binding to RanGTP with high affinity. These conformational changes result in the release of PY-NLS-bearing cargo (Figure 1B) [39,94,95]. Many RBPs bearing PY-NLS that would normally be unable to overcome an NPC’s permeability barrier can be transported into the nucleus through this Ran-regulated NCT (Table 1).

### 2.2. Nuclear Export of Proteins with Nuclear Export Signal by Exportin

A nuclear export signal (NES) is a short signal peptide of four to five hydrophobic amino acids in the proteins that directs the protein out of the nucleus and is recognized by exportins. An NES affects several vital functions of the cell, including transcription and nuclear export [96] A typical NES has an ΦXXXΦXXΦXΦ sequence. Φ is a hydrophobic amino acid; frequently it is leucine, although it can be valine, isoleucine, phenylalanine, or methionine; X can be any other amino acid in the NES sequence [39,45]. NES classes 1a, 1b, 1c, 1d, 2, 3, 1a-R, 1b-R, 1c-R, and 1d-R are described by 10 consensus patterns (Table 2) [97]. All 10 classes of NES bind to the hydrophobic pockets of CRM1 [97,98]. 

The chromosome region maintenance 1 protein (CRM1) or Exportin-1 (Xpo1) is the leading karyopherin that helps with the export of most protein cargos that have a leucine-rich NES (Table 3) [96]. CRM1 binds to cargo in complexes with Ran-GTP inside the nucleus and transports the complex across the NPC. The RanGTP-RanGDP gradient enforces directionality. In the cytoplasm, RanGTP hydrolysis to RanGDP by Ran, with its GTPase activity activated by RanGAP and RanBP1, disassembles the CRM1-cargo complex and releases the cargo (Figure 1B) [99]. Then, CRM1 is recycled to the nucleus, where it can bind to the cargo and RanGTP again for another round of export. On the other hand, RanGDP is cycled back to the nucleus separately, where it is switched back to RanGTP by GEF.

The roles of CRM1 in the nuclear export of TDP-43 [106,107] and FUS [108] have been explored [109]. Computationally, using NES prediction tools, NES finder 0.2 and NetNES 1.1 server, two putative CRM1-dependent NESs were identified in both TDP-43 and FUS [109]. However, the nuclear export of TDP-43 and FUS was independent of CRM1, as confirmed by CRM1 knockdown and CRM1 inhibition assay [109]. Furthermore, TDP-43 and FUS can be exported independently from other export pathways, including Exportin-5 (XPO5) and the mRNA export machinery. Instead, TDP-43 and FUS can leave the nucleus by passive diffusion. In addition, newly synthesized RNA retains TDP-43 in the nucleus and limits its diffusion across the NPCs into the cytoplasm [109]. 

## 3. LLPS of NIR Cargoes Implicated in Neurodegenerative Diseases

Our understanding of membraneless organelles has increased with developments in the field of liquid–liquid phase separation research. LLPS is involved in the formation of many membraneless organelles, such as P bodies, stress granules, nuclear Cajal bodies, nucleolar body, nuclear speckles, and carboxysomes [16,110,111,112]. There is a growing interest in identifying the key factors and conditions responsible for phase separation and formation of these biological condensates, as they are involved in different cellular processes. Modular binding domains and intrinsically disordered regions (IDRs) in protein can drive phase separation, due to their multivalency and flexible interaction modes under physiological conditions [113,114]. Within IDRs, residues exhibiting adhesive properties due to π–π stacking, cation–π interactions, or charge–charge interactions are classified as stickers [115]. The valency and patterning of stickers determine IDR phase behavior. Spacer residues are sequences between stickers; they regulate the material properties of condensates [115,116]. 

NIR cargos, such as FUS, TDP-43, and hnRNPA1, which are IDR-containing RBPs, can undergo liquid–liquid phase separation and are involved in different neurodegenerative diseases [16,117,118]. IDRs can transition between multiple material states on the soluble–phase separated liquid–hydrogel–amyloid spectrum [119]. Factors that alter multivalent interactions, such as RNA, crowding agents, protein concentrations, temperature, pH, and salt content, can affect droplet formation of these IDR-containing proteins [116]. In addition, post-translational modifications, including phosphorylation, methylation, ubiquitination, and sumoylation, can change the strength and valency of interactions, thus affecting the LLPS of proteins with IDR [116]. The proteins that undergo phase separation are discussed in the following section.

### 3.1. FUS

Fused-in sarcoma (FUS) is a RNA-binding protein that plays a role in RNA transcription, splicing, transport, and translation [120]. FUS plays a role in the dendritic maturation and complexity of mouse hippocampal neurons by transporting mRNA to dendrites [121]. FUS protein contains 526 amino acids, and the encoding gene is located on the chromosome number 16 [122]. It comprises an N-terminal prion-like domain (aa 1–267) that contains the RGG1 domain (aa 165–267), the RNA recognition motif (RRM) (aa 285–371) followed by the RGG2 domain, the zinc-finger domain, the RGG3 domain, and a PY-NLS signal at the C-terminus [123]. Mislocalization and cytoplasmic aggregation of FUS have been observed in ALS and FTD patients [124,125]. FUS has been reported to undergo LLPS in vitro [18]. Arginines and tyrosines in the disordered regions of FUS have been identified as stickers that drive FUS LLPS [117]. Glycines are spaces that tune the material state of FUS droplets [117]. In addition, the RNA-binding domains, including RRM, the zinc finger domain, and the RGG domains, can play role in regulating FUS LLPS through interaction with different RNA molecules [126]. Further, RNA concentration, other factors such as the concentration of FUS, and salt can alter the phase separation of FUS [14]. Post-translational modification, such as serine phosphorylation and arginine-methylation in the N-terminal PLD of FUS, can disrupt LLPS [127,128,129,130].

### 3.2. TDP-43

TDP-43 has been identified as one of the major disease proteins aggregating in ALS and FTD patients [6]. It is a DNA/RNA binding protein involved in different cellular processes, such as transcription, translation, mRNA transport, mRNA maturation and stabilization, microRNA, and long non-coding RNA processing [11]. Like many other RBPs, TDP-43 undergoes phase separation through transient intermolecular interactions in the C-terminal prion-like domain (aa 276–414) [3]. One study reported that W334, a tryptophan residue in the α-helical region (aa: 320–340), is required for phase separation in the TDP-43 prion-like domain [3,131]. In addition, the (G/S)-(F/Y)-(G/S) motifs in the C-terminal prion-like domain of TDP-43 promote its phase separation through transient interactions [3]. Salt and RNA concentrations affect the phase separation of the C-terminal region of TDP-43 [14,132]. A low RNA-to-protein ratio promotes the phase separation of TDP-43 in vitro [14]. ALS-linked TDP-43 mutations, such as A321G, Q331K, and M337V, decrease its phase separation ability and increase aggregation [18,132,133]. Interestingly, in vitro studies demonstrated that poly (ADP-ribose) (PAR) promotes the LLPS of TDP-43 by binding to PAR-binding motifs in the NLS of TDP-43 [134]. Recently, it was observed that arginine-rich dipeptide repeats, such as poly-GR and poly-PR can promote phase separation of TDP-43 [135]. Interestingly, it is speculated that the neuron-specific high expression level, longer half-life, and cytoplasmic accumulation of TDP-43 could explain the selective vulnerability of motor neurons in ALS [136].

### 3.3. hnRNPA1

hnRNPA1 is a 320 amino acid long protein and a member of heterogeneous nuclear ribonuclear protein (hnRNP) family [137]. It comprises two folded RRMs in the N-terminal domain and a C-terminal low-complexity domain [16]. It has a PY-NLS signal at the C-terminal domain, which helps in shuttling between cytoplasm and nucleus [138,139]. hnRNPA1 plays multiple roles in gene expression and RNA processing [140,141,142,143,144,145]. Mutations in the LCD domain of hnRNPA1 lead to ALS and multisystem proteinopathy (MSP) [15]. Both diseases are characterized by the accumulation of hnRNPA1 into stress granules and its deposition as solid deposits to cause neurodegeneration [15]. The low-complexity domain of hnRNPA1 is sufficient to induce its LLPS [146]. In addition, multiple types of interactions, such as electrostatic interactions and hydrophobic interactions, contribute to LLPS of hnRNPA1 [16,147]. In the presence of RNA, both the RRM and LCD domains can facilitate LLPS of hnRNPA1 [146]. Further, crowding agents such as Ficoll and PEG, NaCl salt concentration, and increased concentration of hnRNPA1 can affect its phase separation [16]. The disease-causing mutant of hnRNPA1_D262V_ is more associated with stress granule assembly [15]. The LLPS of hnRNPA1_D262V_ mutant can promote its amyloid-like aggregation [16].

## 4. Impaired Nucleocytoplasmic Transport in Neurodegenerative Diseases

**Figure 2:**
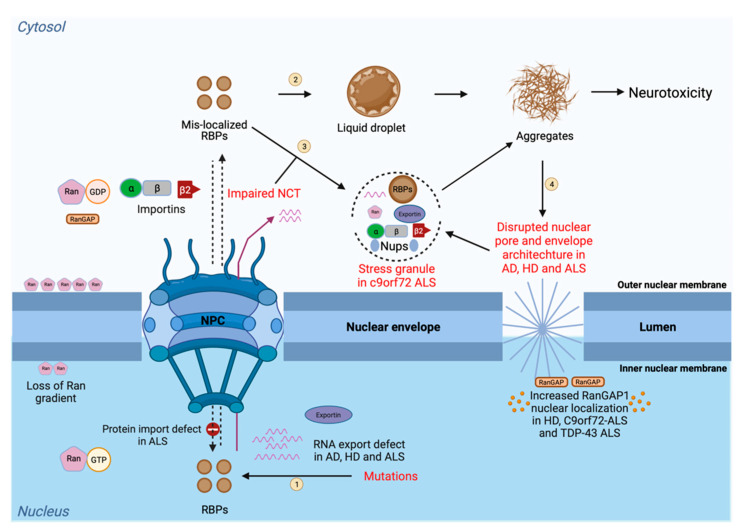
Disease-linked defects in the NCT. Protein transport (import and export) and RNA export are tightly regulated processes through the nuclear pore that depend on different NCT elements, including nucleoporins (Nups), importins, exportins, and transport factors, together with Ran and its regulatory components. The steps in the process are as follows: (1) Mutations in RNA-binding proteins lead to its mislocalization to the cytoplasm from the nucleus; (2) In the cytoplasm, these mislocalized RBPs can undergo liquid–liquid phase separation and aberrant phase transition to form irreversible aggregates; (3) RBPs can also participate in membraneless organelles, such as stress granule, which can further form irreversible aggregates. Impairments in the NCT also lead to the mislocalization of RBPs in the cytoplasm and cause their accumulation in phase-separated biocondensates which can further convert into irreversible toxic aggregates leading to neurotoxicity; and (4) In the presence of toxic aggregates generated from RBPs, the nuclear pore integrity and the nuclear membrane morphology are compromised, leading to the uncontrolled transport of proteins and RNAs. Due to this, the Ran gradient becomes impaired and causes an abnormal accumulation of Ran in the cytoplasm and abnormal RanGAP localization in the nucleus. Mislocalization of RBPs causes sequestering of NCT factors, such as importins and nucleoporins, into the cytoplasm-forming SG. SG formation further disrupts the process, due to the generation of neurotoxic aggregates. This figure was created with BioRender.com.

A shared pathological feature of multiple neurodegenerative diseases is the presence of large intracellular or extracellular inclusions in the brain of diseased patients that cannot be degraded by normal mechanisms. Protein aggregates could induce nucleocytoplasmic defects by sequestering components of the NPC or directly blocking the NPC, leading to protein mislocalization and aggregation [27,148,149]. Moreover, mislocalized proteins that aggregated in different compartments (e.g., nuclear aggregates and cytoplasmic aggregates) can have different toxic consequence in neurons, as shown in a mouse model [150]. Therefore, it is proposed that protein mislocalization, aggregation, and nucleocytoplasmic transport defects form a feed-forward loop, and together they contribute to cellular toxicity in neurodegenerative diseases (Figure 2). Indeed, impaired nucleocytoplasmic transport mechanisms are implicated in ageing and in neurodegenerative diseases such as amyotrophic lateral sclerosis (ALS), frontotemporal dementia (FTD), Huntington disease (HD), Alzheimer’s disease (AD), Parkinson’s disease, and tauopathies [151]. Disease-linked mutations and the abnormal aggregation of proteins implicated in these neurodegenerative diseases can cause impairments in NCT by (1) changing the nuclear membrane structure, (2) mislocalizing the Nups, Ran, and its regulatory components in the cytoplasm, and (3) blocking the nuclear export [152,153,154,155,156,157,158,159,160,161,162]. The following section discusses various neurodegenerative diseases and their NCT defects (Figure 2).

### 4.1. Impaired NCT in ALS and FTLD

ALS is a late-onset, relentlessly progressive, prototypic age-dependent neurodegenerative disorder caused by the loss of motor neurons, leading to paralysis and death [11,163]. The disease leads to lethal consequences after 3 to 5 years of symptom inception and is marked by progressive muscle weakness, followed by respiratory failure [164]. Out of every 100,000 individuals worldwide, approximately five are likely to be affected by the disease. ALS cases are categorized into sporadic (sALS) and familial (fALS); 90% to 95% of the cases belong to the former class [11,165,166]. 

FTLD is a progressive neurodegenerative disease associated with neuronal degeneration in the frontal and temporal lobes, with neuronal intranuclear and cytoplasmic inclusions [167,168]. FTLD is the second-most abundant dementia before the age of 65, after Alzheimer’s disease. Out of 100,000 individuals worldwide, approximately 15 to 22 are likely to be affected by the disease [169]. It is characterized by significant behavioral changes, personality changes, impaired language skills, and presenile dementia [167]. 

Both diseases are heterogeneous and share some clinical, neuropathological, and genetic features [170]. In addition to their specific characteristic features, the main common clinical features in patients are subclinical frontal dysfunctioning, language impairment, and progressive aphasia phenotypes [171,172,173]. In addition to clinical overlap, both diseases share features at the molecular level as well; different studies have led to the identification of TDP-43, FUS, SOD1, and dipeptide repeats in C9orf72 as the major aggregating proteins involved in ALS and FTLD patients [6,124,174,175,176,177,178,179]. Further, the accumulation of the microtubule-associated protein tau is observed in FTLD patients [177]. A number of mutant genes have been recently identified as causing ALS and FTLD [154,168,180,181,182,183,184,185,186,187,188,189,190,191,192,193,194]. Various pathophysiological mechanisms, such as dysregulation in protein homeostasis due to compromised protein degradation pathways (e.g., perturbed ubiquitin-proteasome system pathway, disturbed autophagy, and inhibition of endocytosis), abnormal stress granule assembly, glutamate excitotoxicity, dysregulation in metal ion homeostasis, and defects in nucleocytoplasmic transport, are involved in ALS and FTLD [195,196,197,198]. 

Different reports indicated that impairment in the NCT is one the major mechanisms of ALS and FTLD pathology. Cytoplasmic TDP-43 aggregates were found to be accumulated with many Nups and transport factors, suggesting their role in disrupting the NCT [149,199]. The mislocalization and aggregation of TDP-43 also leads to interruption in the normal nuclear staining of Nup62 and Kapβ1 in spinal motor neurons of sporadic ALS patients [156]. A reduction in the Nup protein levels was observed in the TDP-43 knockout cells, leading to an anomaly in nuclear pore morphology [157]. Moreover, the loss of impβ1 immunoreactivity and ruffled nuclear morphology when staining with Nup62 and Nup153 antibodies were reported for sporadic ALS tissue [148,156,200,201]. 

The GGGGCC (G4C2) hexanucleotide repeat expansion (HRE) in C9orf72 is the most common genetic cause of fALS and fFTD [202,203]. G4C2 repeats can trigger disease pathology through three different mechanisms, including loss of C9orf72 protein function [175,202,204,205,206,207], the formation of RNA foci [208], and the production of dipeptide repeat proteins (DPRs) via repeat-associated non-AUG (RAN) translation [209,210,211]. The C9orf72 G4C2 repeat RNA can interact with RanGAP1, causing it to mislocalize and disrupt the Ran gradient [212]. G4C2 repeat RNA can also reduce the expression of the transmembrane nucleoporin POM121, which in turn can reduce the expression of seven additional nucleoporins, ultimately affecting Ran localization and cellular toxicity [213]. In addition to G4C2 repeat RNA, DPR accumulation in ALS and FTLD patients can result in nucleocytoplasmic transport defects [214,215]. Poly-glycine-alanine (GA) and poly-glycine-arginine (GR) from the sense strand, poly-proline-alanine (PA) and poly-proline-arginine (PR) from the antisense strand, and poly-glycine-proline (GP) from both strands are the five DPRs currently known to form via RAN translation of G4C2 HRE [216,217]. Arginine-rich poly-PR peptides are reported to disrupt the NCT by masking the FG repeats of Nups [218]. Poly-GA aggregates affect the nuclear import of TDP-43 by impairing the importin-α/β-dependent pathway, but the mechanism needs more investigation [219]. Together, G4C2 repeat RNA and DPRs accumulation cause defects in the NCT by affecting the activity and localization of Nups, karyopherins, and RanGAP in ALS and FTLD patients [212,214,215]. Moreover, recently arginine-rich dipeptide repeats have been shown to interact directly with NIRs to inhibit the nuclear import [220]. 

Further, mutations in other proteins implicated in ALS, such as senataxin and Vesicle-associated membrane protein-associated protein B/C can cause impairment in the NCT [153,154,155]. Mutations in small acting-binding protein profilin 1, which is implicated in ALS, causes defects in the NCT and further affects the normal function of ALS-relevant RBPs, leading to motor neuron dysfunction [152]. Mutations in the human cytoplasmic Nup Gle1, which is important for the nuclear mRNA export, have also been identified as causing ALS [221,222]. Another study reported that tau mutation in FTLD causes a hyperphosphorylation and mislocalization of tau in cortical neurons, which leads to a defect in the NCT by mis-shaping the nuclear membrane [223]. In conclusion, a great deal of evidence has suggested that NCT dysregulation is a major pathogenic driver in ALS and FTLD-linked neurodegeneration. 

### 4.2. Impaired NCT in Alzheimer’s Disease

Alzheimer’s disease is a chronic neurodegenerative disease with well-defined pathological mechanisms. It contributes to 60% to 80% of dementia cases [224]. Neuritic plaques that are formed due to an accumulation of the amyloid-beta peptide (Aβ) in brain tissues and neurofibrillary tangles (NFTs) formed due to an accumulation of the microtubule-associated tau protein in neurons are the major pathological hallmarks of AD [4]. The proteolytic cleavage of the amyloid beta precursor protein (APP) leads to the generation of Aβ40 and Aβ42 peptides [225]. Overproduction of Aβ42 peptides due to genetic, age-related, or environmental factors leads to their accumulation as insoluble oligomers, protofibrils, and aggregates. Aggregated Aβ species can further convert into senile and neuritic plaques in the brain regions [225]. In addition, abnormal accumulations of Aβ can further result in the phosphorylation and aggregation of tau as NFTs [226,227]. These processes can cause activation of neurotoxic events that ultimately lead to cytoskeletal changes, neuronal dysfunction, and cellular death [227]. 

Recently, disruption of neuronal NCT was observed as one of the major pathological features involved in AD [27]. Using advanced techniques, such as immunohistology and electron microscopy, prominent irregularities and invaginations have been observed in neuronal nuclear envelope in the post-mortem brain tissues of AD patients [27,158,159,223]. Multiple studies have suggested that nuclear pores, Nups, and other transport factors are disrupted in AD, which can dysregulate the NCT [26]. For example, NTF2, a RanGDP transporter and a key NCT factor, was found to have abnormal cytoplasmic accumulation in the hippocampal neurons of AD brains, indicating impaired NCT [159,228]. Another study identified the abnormal localization of importin α1 to hirano bodies in AD hippocampal neurons [229]. In conclusion, many different studies on brain biopsies of AD patients have revealed that impairment of the NCT is one of the major pathological causes of AD. 

The mechanism of NCT impairment in AD has been studied in the context of Aβ and tau aggregates. A study demonstrated that oligomeric Aβ species can reduce the Ran expression level and disrupt the nucleocytoplasmic transport in AD models [230]. Additional evidence exists for the mechanism of NCT impairment involving tau. Intranuclear tau protein is involved in multiple cellular functions, such as DNA integrity maintenance [231,232,233], DNA repair [233,234], gene regulation [235], ribosomal gene translation, and assembly [26,236]. In AD patients, significant amounts of extranuclear tau are present in the somatodendritic compartment and interact with the outer nuclear envelope [26]. The accumulation of tau in the somatodendritic compartment can raise tau concentration in the perinuclear space and decrease the rate of nuclear import and export [27]. Pathological tau can affect the nuclear architecture by causing an abnormality in the nuclear membrane and the clumping of nuclear pores in NFT-neurons [160,161,227]. Nuclear envelope invagination caused by mutant tau leads to a toxic accumulation of mRNA [158]. In addition to changing the nuclear membrane structure, cytoplasmic tau protein has the potential to directly interact with NPCs [27]. As a result, abnormal NPC distribution and FG-Nups accumulation in the cytoplasm have been observed in hippocampal neurons and NFT-neurons, respectively, in post-mortem AD brains [27]. Even in tangle-free neurons, phospho-tau accumulation has been identified at the nuclear membrane [237]. 

### 4.3. Impaired NCT in Huntington’s Disease

Huntington’s disease (HD) is a rare, inherited, and progressive neurodegenerative disease caused by CAG-repeat expansion in exon 1 of the huntingtin gene, leading to the expression of mutant huntingtin (Htt) protein with expanded polyglutamine (polyQ) repeat [238,239]. Htt protein has an internal NLS and NES sequence that allows it to shuttle between the nucleus and the cytoplasm [240,241,242]. Under disease conditions, the aggregation of PolyQ-Htt has been observed in both the nucleus and the cytoplasm of neurons of the striatum, cortical, and hippocampal regions of the brain [238,243,244,245]. These aggregates are pathological and can induce neurotoxicity [162,245]. Multiple reports indicated that nucleocytoplasmic compartmentalization is severely disrupted in different models of repeat expansion diseases, including HD [162,212,214,218,222,246,247,248,249,250,251,252,253]. A proteomics study demonstrated the sequestration of different nuclear pore complex proteins, such as Nup62, Nup153, Nup214, and Nup358, within intracellular polyQ-Htt aggregates [254]. One study revealed the interaction of Nup62 and RanGAP1 with intranuclear polyQ-Htt inclusions across different models, such as transgenic mouse, drosophila, primary neurons, HD patient-derived iPSC neurons, and post-mortem human HD brain regions [245]. In mouse and cell models of HD, perinuclear inclusions of mutant Htt disrupt the nuclear membrane, causing cell-cycle re-entry and striatal cell death [162]. The mislocalization of NCT factors, such as Gle1 and RanGAP1, has been observed in the presence of polyQ-Htt aggregates in mouse models and in HD patients, resulting in a disturbed Ran gradient [255]. In certain HD animal models and human patients, the impairment of nuclear export leads to nuclear accumulation of polyA-mRNA [255]. Thus, mutant Htt-mediated NCT defects are a common phenotype in HD.

### 4.4. NCT Impairments in Ageing

The prevalence of most neurodegenerative diseases, including AD, PD, and ALS, rises with age. Age-related neurodegenerative disorders have few or no effective therapies, and they tend to proceed in an irreversible way, resulting in high economical and human consequences [256]. Normal physiological ageing can also gradually impair the NCT due to the progressive failing of protein homeostasis that is required for the proper assembly, repair, and maintenance of NPCs in regulating normal cellular health [257,258]. Compromised NPC quality control in mitotic cells can lead to impairment in the shuttling of transcription factors [259]. Moreover, one study showed that oxidative stress in aged cells can damage the scaffolding Nups, which can further lead to a leaking of cytoplasmic proteins into the nucleus by increasing the nuclear permeability [260]. 

Some differentiated cells, such as muscle fibers and neurons, have long-lived scaffold nucleoporin proteins, such as Nup93 and Nup107 [260]. These long-lived nucleoporins may be more susceptible to protein degradation as cells age and no longer remain bound to the NPC. Specifically, Nup93 functions as a link between the Nup107/160 scaffold and the FG-nucleoporins from the central channel of the NPC. In the absence of Nup93, FG-nucleoporins are lost, and the permeability barrier deteriorates [260]. In conclusion, deficits in the NCT are also related to normal ageing. 

### 4.5. Selective Neuronal Vulnerability to Nucleocytoplasmic Transport Deficits in Neuro-Degenerative Diseases

The aforementioned neurodegenerative diseases target different regions of the brain, but they all selectively target a subpopulation of neurons, leading to the progressive failure of defined nervous system regions. Selective neuronal vulnerability is a shared property of these neurodegenerative diseases, but the basis of such selective neuronal vulnerability has remained elusive. Interestingly, different neuronal cells showed different susceptibility to nucleocytoplasmic transport defect [261]. This different susceptibility to nuclear pore deficits may be a result of cell type specific NPCs, differential expression of protective protein, or selective transport of proteins and mRNAs in different neurons [261,262]. Therefore, it is tempting to speculate that different susceptibility to NCT defect may contribute to the selective vulnerability of certain neurons in neurodegenerative diseases. Indeed, several pieces of evidence suggest that selective neuronal vulnerability to NCT deficits might be linked to selective susceptibility of neurons. For example, striatal Medium Spiny Neurons (MSN) are particularly vulnerable at early stages of HD progression. In a study using iPSC-derived neurons as HD models, it is shown that MSNs are more susceptible to nuclear pore deficits than other neural cell types tested and may contribute to the vulnerability of MSNs in HD [263]. Moreover, in ALS, upper motor neurons (UMN) and lower motor neurons (LMN) are specifically targeted but not equally affected. For example, some LMN subtypes are relatively resistant to neurodegeneration, including oculomotor neurons and Onuf’s nuclei MNs. Interestingly, lamin B1 whose expression level is linked to nucleocytoplasmic transport defect, was upregulated in oculomotor neurons compared to hypo-glossal MNs and spinal cord MNs [264]. It is therefore possible that nucleocytoplasmic transport defect is more susceptible to specific MN populations. However, further work is necessary to examine this possibility.

### 4.6. Restoring Nucleocytoplasmic Transport by Small Molecules for the Treatment of Neurodegenerative Diseases

Regardless of the significant evidence of NCT difficulties in ALS, FTD, HD, and AD, there is currently no treatment option for neurodegenerative disorders that targets nuclear transport impairments. Finding new approaches to repair NCT disruption in neurodegenerative proteinopathies is a tantalizing new potential approach for avoiding neuronal death in these disorders, but it is also a daunting task. The tremendous molecular and structural complexity of the NPC, as well as the relevance of NCT for practically all cellular activities, present major obstacles in designing treatment techniques. 

NPC disturbances frequently result in an imbalance in the nucleocytoplasmic gradient of NTFs, transcription factors, nuclear proteins, and RNA, which can be partially reversed by boosting or inhibiting nuclear import or export [265,266]. CRM1 is the primary receptor for protein export out of the nucleus [96,97]. Selective inhibitors of nuclear export (SINE), such as KPT-350, KPT-335, and KPT-276, were predicted using a structure-based drug design for CRM1 and were found to be successful in preclinical models [148,212,267,268]. However, due to capability of CRM1 in transporting a wide spectrum of molecular cargos out of the nucleus, off-target effects and potential toxicity are still concerns when targeting this pathway. Phase 1 trials of another CRM1 inhibitor were recently initiated to explore the safety and positive benefits of CRM1 inhibition vs. the off-target effects in ALS patients to determine whether inhibiting nucleocytoplasmic export is adequate to prevent abnormal neuronal death in humans [269]. DPRs can disrupt multiple nucleocytoplasmic transport pathways [270]. In a new study using a cell-based phenotypic screen, several commercially available compound libraries containing 2714 compounds were evaluated to counteract the toxicity caused by PR-50 and its effect in disrupting the nucleocytoplasmic transport pathways [270]. Several epigenetic protein inhibitors (such as HDAC inhibitors, EZH1/EZH2 inhibitors, HAT activators, and SIRT1 activators) were discovered as possible hits, and they improved cell viability and restored nucleocytoplasmic transport in PR-50-expressing cells in addition to a compound that is already in clinical trials for the treatment of ALS (Na-4-phenylbutyrate) [270].

## 5. Effect of NIRs on LLPS of NIR Cargoes

NIRs employ nuclear-localization signal of polypeptide cargo in the cytoplasm for their transport to the nucleoplasm. Through binding to NLS, NIRs have the potential to inhibit and reverse the aberrant phase transitions of several intrinsically disordered RNA-binding proteins with prion-like domains, such as FUS, TDP-43, hnRNPA1, hnRNPA2, EWSR1, and TAF15, as well as their disease-linked mutant variants [22]. Higher expression levels of NIRs can modify the deteriorating phenotypes associated with atypical aggregations of RBPs [22]. For example, Kapβ2 can tightly bind to the C-terminal PY-NLS region of FUS to inhibit and reverse its liquid–liquid phase separation and aggregation [22,23,24,25]. An NMR study showed that, in addition to PY-NLS, there are multiple Kapβ2 binding sites of different strength distributed throughout the FUS protein [23]. Most of these regions are involved in the LLPS of FUS [23]. Kapβ2-binding to these regions can disrupt the self-association of FUS, thus preventing the LLPS of FUS [23]. Kapβ2 can also reduce the association of FUS with stress granules (SG) without affecting the SG biogenesis [22,24]. Further, it was reported that elevating Kapβ2 expression levels can relieve FUS toxicity by reversing its aberrant aggregation and by restoring its nuclear localization [22]. Mutations in the PY-NLS of FUS causes the most severe type of ALS. For example, ALS patients with FUS-P525L missense mutation, and FUS-R495X nonsense mutation experience juvenile ALS [271,272]. These mutants cause the FUS variants to mislocalize in the cytoplasm and have decreased the binding affinity with Kapβ2, compared with that of wild-type FUS [108,273]. Despite the absence of PY-NLS, FUS-R495X maintains connections with Kapβ2, which suppresses its LLPS [274]. The RGG2-ZnF-RGG3 region of FUS-R495X binds to the PY-NLS binding site of Kapβ2 to mediate this interaction [274]. Arginine methylation in FUS regulates its nuclear localization. Inhibition of arginine methylation leads to increased accumulation of FUS-R495X and the RGG2-ZnF-RGG3 segment in the nucleus [275]. Kapβ2 can also prevent the phase separation of hypomethylated FUS, which is commonly observed in ALS patients [25,275,276].

In addition to Kapβ2 (which is also called TNPO1), a few other nuclear import receptors, including transportin-3, importin β, importin 7, or the importin β/7 heterodimers, directly bind to FUS via its RG/RGG motifs [277]. In addition, trasnportin-3 has shown its potential to import FUS into the nucleus in digitonin-permeabilized HeLa cells [277]. The interaction of FUS with these NIRs can suppress and reverse FUS phase separation and sedimentation, indicating their chaperone activity [277]. These NIRs can also reduce the sequestration of FUS into stress granules [277]. Other NIRs, such as Impβ, Imp5, and Imp9, can also bind to the RGG regions of the FUS-R495X and mediate their nuclear import [274]. Another NIR, Impα, in combination with impβ, can inhibit the aggregation of TDP-43 [22]. 

RAN translation of the C9orf72 HRE produces arginine-rich DPRs (R-DPRs) (i.e., poly (GR) and poly (PR)), which are the most toxic of the five DPRs generated in neurons [278]. These R-DPRs can directly interact with TDP-43 and other RBPs and alter their LLPS or aggregation behavior [135]. NIRs have a strong binding affinity toward R-DPRs [135]. Several NIRs are identified as modulators of R-DPRs’ toxicity in c9ALS/FTLD models [214,246,278,279]. Moreover, both Kapβ1 and Kapβ2 inhibit the RNA-stimulated condensation of poly (GR) [220]. In addition, elevated levels of Kapβ1 and Kapβ2 can shield R-DPRs and prevent their pathological interaction with TDP-43 or other RBPs [220]. Other DPR, such as poly (GA) and chimeric DPR species GA:GP, can also form cytoplasmic inclusions and affect the TDP-43 nuclear import in c9ALS/FTLD [219,280]. Poly (GA) expression induced robust TDP-43 cytoplasmic mislocalization in hippocampal neurons and overexpression of NIRs, such as Impα3 and Impα4, restored TDP-43 to the nucleus [281,282]. Thus, NIRs function broadly to reverse the aberrant phase transitions of RBPs that are involved in human neurodegenerative diseases.

## 6. Enhancing Chaperone Activities of Nuclear Import Receptors

**Figure 3:**
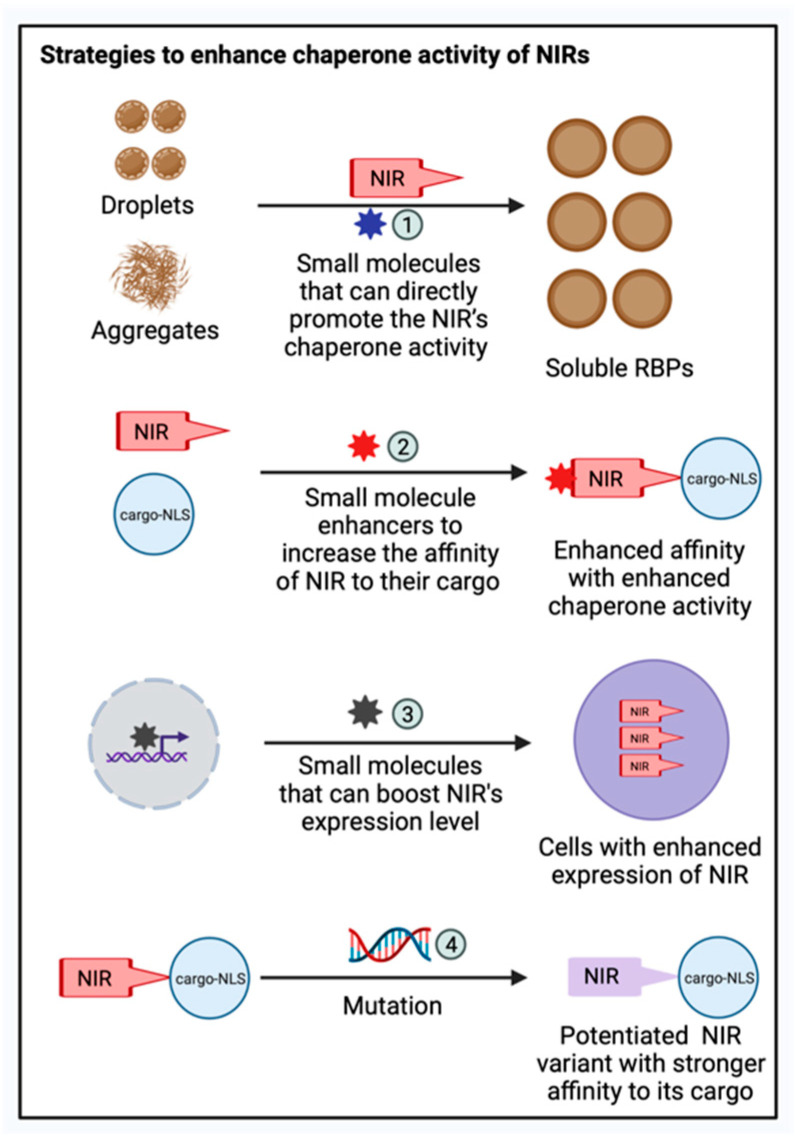
Therapeutic strategies to enhance the chaperone activity of NIRs. Various therapeutic strategies can be applied to enhance the chaperone activities of NIRs against mislocalized and aggregated RBPs: (1) Screening for drug-like molecules that can improve NIR’s ability to prevent or reverse RBP fibrillization; (2) Designing small molecule enhancers that can increase the affinity of NIRs to their cargo; (3) Screening small molecules that can boost the expression level of NIRs in cell-based models; and (4) Exploiting complex structure information of NIR bound to their cargo to develop potentiated NIR variants with stronger affinity towards their cargo. This figure was created with BioRender.com.

The chaperone and disaggregase activities of nuclear import receptor family members have become an attractive target for therapeutic development in conditions involving the protein aggregates. Screening for drug-like molecules that improve NIRs’ ability to prevent or reverse RBP fibrillization will be very interesting (Figure 3). Different classes of small molecules can be designed or screened. For example, a high-throughput screening strategy can be employed to identify molecules that can directly promote NIRs’ activities on disaggregating preformed RBP fibrils (Figure 3). Alternatively, small molecule enhancers that can increase the affinity of NIRs to their cargo can be designed, based on the hypothesis that enhanced affinity results in an enhanced chaperone activity of NIRs (Figure 3). 

Moreover, a chaperone’s activity in cell can be boosted by small molecules that can boost their expression level, which can be screened using cell-based models (Figure 3). In addition to small molecule drugs, engineered NIR variants with improved activity are attractive strategies to enhance the chaperone activities of NIR. For example, there are multiple structures available for Kapβ2 bound to their cargo, including FUS, hnRNPA1, and ALS-linked FUS variants [78,92,274,283]. This information can be exploited to develop potentiated Kapβ2 variants with stronger affinity toward their cargo (Figure 3). Such potentiated variants can also be identified using an unbiased high-throughput screening approach that screens the entire protein sequence space, as shown for another protein disaggregase, Hsp104 [284,285,286,287,288,289]. The delivery of these potentiated Kapβ2 variants could be achieved by adeno-associated viruses (AAVs) or other gene-delivery methods.

## 7. Conclusions

Many proteins rely on their intracellular localization to function properly. Therefore, NCT is critical for a wide range of physiological and pathological processes. Defects in NCT have been associated with several neurodegenerative disorders characterized by protein mislocalization and the formation of toxic protein aggregates resulting from aberrant protein phase transition. These aggregates can trigger more alterations in the NCT machinery, either by causing morphological changes in the nuclear pore complex and nuclear membrane or by the mislocalization of NCT components, including Nups, importins (NIRs), exportins, and other regulatory factors. Although NCT impairments have been linked to several neurodegenerative disorders and ageing, no therapeutic strategies are available. Nuclear import receptors can act as a chaperone and have the potential to inhibit or reverse the aberrant phase separation and toxic aggregation of different RBPs. They can also effectively restore the nuclear localization of mislocalized RBPs to their normal function. Thus, potentiating NIRs’ activity using small molecules or genetic approaches to prevent the mislocalization and aberrant phase transition of RBPs involved in disease pathology may be a promising technique.

## Figures and Tables

**Table 1:** Different NLS sequences recognized by different importins.

Category	Type	Protein	NLS Amino Acid Sequence	Importins	Reference
Classical nuclear localization signal (cNLS)	MP NLS	VACM-1/CUL5	PKLKRQ	Importin α/β1	[52]
		CXCR4	RPRK		[53]
		VP1	RRARRPRG		[54]
		c-myc	PAAKRVKLD/RQRRNELKRSF		[55]
		Polyoma large T-Ag	VSRKRPRP		[56,57]
		Hepatitis D virus antigen	EGAPPAKRAR		[58,59]
		NF-κB p50	QRKRQK	Importin α3 & Importin α4	[60,61,62]
		NF-κB p65	EEKRKR		[62,63]
		SV40 T antigen	PKKKRKV	Importin α	[64,65]
		murine p53	PPQPKKKPLDGE		[66,67]
	BP NLS	Nucleoplasmin	KRPAATKKAGOAKKKK	Importin α/β1	[64]
		ING4	KGKKGRTQKEKKAARARSKGKN		[68]
		IER5	RKRCAAGVGGGPAGCPAPGSTPLKKPRR		[69]
		ERK5	RKPVTAQERQREREEKRRRRQERAKEREKRRQERER	Importin 7	[70,71]
		53BP1	GKRKLITSEEERSPAKRGRKS	Importin α	[72]
		Rat glucocorticoid receptor	YRKCLQAGMNLEARKTKKKIKGIQQATA	Importin 7 & Importin α/β1	[73,74]
		RCC1	MSPKRIAKRRSPPADAIPKSKKVKVSHR	Importin α3	[75]
Non-classical nuclear localization signals (ncNLS)	PY-NLS	Hrp1	RSGGNHRRNGRGGRGGYNRRNNGYHPY	Imp β2	[76]
		UL79	TLLLRETMNNLGVSDHAVLSRKTPQPY		[51]
		FUS	FGPGKMDSRGEHRQDRRERPY		[77,78]
		hnRNPA1	NNOSSNFGPMKGGNFGGRSSGPYG		[77,78]
		hnRNPA2	NQQPSNYGPMKSGNFGGSRNMGGPYG		[77]
		TAF15	GYGGKMGGRNDYRNDQRNRPY		[77]
		EWS	GGPGKMDKGEHRQERRDRPY		[77]
	Other ncNLS	Pho4	SANKVTKNKSNSSPYLNKRKGKPGPDS	Impβ family member Pse1/Kap121	[79]
		rpL23a	VHSHKKKKIPTSPTFTTPKTLTLRRQPKYPRKSAPRRNKLDHY	Impβ, transportin, RanBP5 and RanBP7	[80]
		PTHrP	GKKKKGKPGKRREQRKKKRRT	Imp β1	[81]
Other types of nuclear localization signals	Putative NLS	PABPN1	None		[47]
	Spatial epitope NLS	STAT1	None		
	Cryptic NLS	FGF2	None		
	Multiple NLS	MSX1	RKHKTNRKPR NRRAKAKR		[82]
		NLS-RARα	RNKKKK RKVIK	Importin α1/β1	[83]

**Table 2:** NES classes with sequence patterns.

Class	Sequence Pattern
1a	ΦXXXΦXXΦXΦ
1b	ΦXXΦXXΦXΦ
1c	ΦXXXΦXXXΦXΦ
1d	ΦXXΦXXXΦXΦ
2	ΦXΦXXΦXΦ
3	ΦXXΦXXXΦXXΦ
1a-R	ΦXΦXXΦXXXΦ
1b-R	ΦXΦXXΦXXΦ
1c-R	ΦXΦXXXΦXXXΦ
1d-R	ΦXΦXXXΦXXΦ

Note: (Φ is Leu, Val, Ile, Phe, or Met, and X is any amino acid).

**Table 3:** Exportins-NES interactions.

Exportin	Type of NES	Protein	NES Sequence	Reference
CRM1/Xpo1	Leucine-rich	CPEB4	RTFDMHSLESSLIDI	[98]
		hRio2	RSFEMTEFNQALEEI	
		Yap1p	SDIDVDGLCSELMAKAK	
		Stat3	RGLSIEQLTTLAEKLL	
		TIS11	MDLSAIYESLMSMSH	
		rZap	SVDVTQKFKYLGTHDR	
		Menin^1^	DSLELLQLQQKLLWLLY	
		Pap1	ESFDIDDLCSKLKNKAK	
		DOK7	ETLQLEKRLSLLSHA	
		hnRNPA1	NQSSNFGPMKGGNFGGRSSGPYGGGGQYFAKPRNQGGY	[100]
		RanBP1	DHAEKVAEKLEALSV	[101]
Exportin2/CAS/Xpo2	-	Imp-α	Conformational or entire Impα	[102]
Exportin-4/Xpo4	-	eIF-5A and other proteins	-	[103,104]
Exportin-5/Xpo5/RanBP21	-	RNA and proteins	Conformational or entire pre-miRNA	[104]
Exportin-6/Xpo6/RanBP20	-	Nuclear export of actin and profilin-actin complexes	-	[104,105]
Exportin-7/Xpo7/RanBP16	-	Proteins	-	[104]
Exportin-t/Xpot	-	tRNAs	Conformational or entire tRNAs	[102]

## Data Availability

Not applicable.
